# The Effects of Low Level Laser Therapy on the Expression of Collagen Type I Gene and Proliferation of Human Gingival Fibroblasts (Hgf3-Pi 53): *in vitro* Study

**Published:** 2013-10

**Authors:** Ali Frozanfar, Mohammad Ramezani, Amin Rahpeyma, Saeedeh Khajehahmadi, Hamid Reza Arbab

**Affiliations:** 1 Oral& Maxillofacial Diseases Research Center, Faculty of Dentistry, Mashhad University of Medical Sciences, Mashhad, Iran; 2 Pharmaceutical Research Center, School of Pharmacy, Mashhad University of Medical Sciences, Mashhad, Iran; 3 Dental Research Center, Faculty of Dentistry, Mashhad University of Medical Sciences, Mashhad, Iran

**Keywords:** Collagen type I, Human gingival fibroblasts, Low level laser therapy

## Abstract

***Objective(s):*** Recent investigations show that both proliferation and secretion of macromolecules by cells can be regulated by low level laser therapy (LLLT). The aim of this study was to determine whether LLLT could induce a bio-stimulatory effects on human gingival fibroblasts (HGF3-PI 53). Therefore, the effect of laser irradiation on human gingival cell proliferation and collagen type I gene expression was studied.

***Materials and Methods:*** HGF3-PI 53 were cultured in 96-well plate and then irradiated with LLLT gallium-aluminum-arsenide (Ga–Al–As), 810 nm, 50 mW diode laser (energy: 4 J/cm^2^) for three consecutive days. The cell proliferation was measured on days 1, 2 and 3 after irradiation with LLLT using MTT assay. Real time PCR analysis was utilized on day 3 to evaluate the expression of collagen type I gene.

***Results*** *:* Evaluation of cellular proliferation, one day after laser treatment showed no difference compared to control group. But on days 2 and 3, significant increase in proliferation was observed in the irradiated cell populations in comparison to the control group. Treatment of HGF3-PI 53 by laser resulted in a significant increase in collagen I gene expression on 3 day.

***Conclusion:*** The results demonstrated that LLLT stimulated human gingival fibroblast proliferation as well as collagen type I gene expression *in vitro*.

## Introduction

Low level laser therapy (LLLT) in oral cavity is used in periodontal and orthognathic surgeries causing acceleration of wound healing, managing chronic mucosal disease, and improving temporomandibular joint disorders. Reducing pain after endodontic and wisdom tooth removal are other clinical applications (1-5). LLLT has achieved acceptance since 30 years ago and its application is more extensive today (6). Improving skin wound repair, bone remodeling, pain reduction and regulating immune responses are attributed to low power laser biostimulator effect (7). Gingival fibroblasts have important roles in gingival wound healing and maintenance, especially after periodontal surgeries or conservative periodontal therapies. These cells produce collagen, elastin, fibronectin, and proteoglycans which have structural roles in gingival connective tissue. Enzyme and enzyme inhibitors as well as growth factors are produced by fibroblasts (8, 9). In this study, the effects of LLL gallium–aluminum–arsenide (Ga–Al–As) on human gingival fibroblasts (HGF3-PI 53) proliferation and collagen type I gene expression were studied.

## Materials and Methods

***Fibroblast cell culturing***

**Figure 1:**
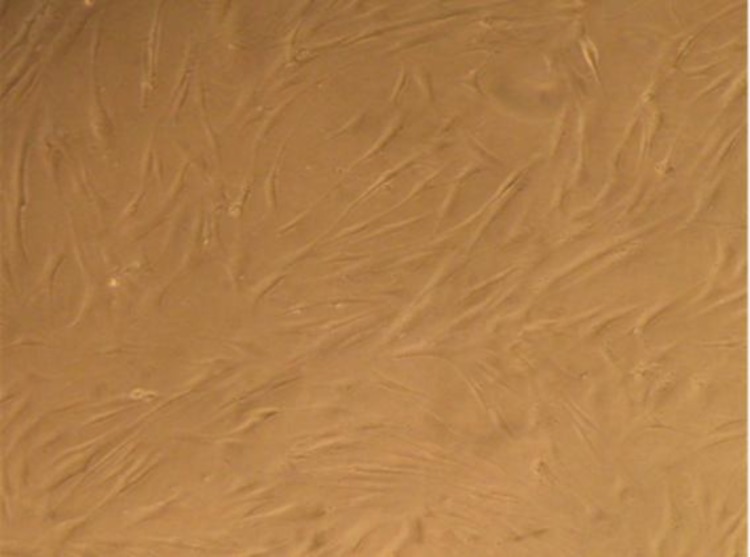
Human gingival fibroblasts growing *in vitro*

Human gingival fibroblasts (HGF3-PI 53 NCBI code C502) were obtained from Pasteur Institute, Iran. Culture process was performed according to the cell culture protocol of American Type Culture Collection (ATCC) (Figure 1). 

The cells were cultured in Dulbecco's Modified Eagle's Medium (Gibco, USA) supplemented with 10% fetal bovine serum (FBS). This medium was also supplemented with 2 mM L-glutamine, 100 U/ml penicillin, and 100 g/ml streptomycin. Culture media was changed every two days. Overlying liquid culture media was removed with micropipettes and 6 ml fresh culture media added to each 25 cm^2^ flask. Cell passage was done. First, cells were washed with buffered PBS followed by addition of 1 ml trypsin solution (0.25 %) for 3 min. Then, FBS (5 ml) was added to neutralize the trypsin. The cells were removed by centrifugation at 1200 rpm. The cells were re-suspended in fresh culture medium and the cell suspension was transferred into three flasks. 

***MTT assay***

HGF3-PI 53 cells were seeded in 24-well plates at an initial density of 5 × 10^3^ cells/well and incubated for 24 hr at 37C under 5% CO_2_ atmosphere prior to exposure to laser irradiation. For cell proliferation assay, 50 µl sterile filtered MTT stock solution (5 mg/ml) in 0.1 N HCl in anhydrous isopropanol was added to each well. After 3 hr, the non-reacted dye was removed by aspiration. The formazen crystals were dissolved in 100 µl /well DMSO. The plate absorbance was read at 570 nm (10).

***Low level laser irradiation***

Infrared laser irradiation was obtained using an 810-nm-emitting gallium–aluminum–arsenide (Ga–Al–As) diode laser (Advanced, Australia) in continuous mode and a maximal output power of 50 mW. The wells were irradiated with laser beam from a 5 mm distance. The output laser beam was directed toward the center of the wells over a 0.4 cm^2^ zone through medium culture. Required irradiation time for delivering an energy density of 4 J/cm^2^, was 32 sec. The laser treatment was performed after 24 hr followed by 48 and 72 hr of incubation. The control group received no laser irradiation but was removed from the CO_2_ incubator for the same period of time as treatment plates.

***Real-time PCR Primer Design***

Total RNA was extracted using the Qiazol (Qiagen, Germany) after 3 days of incubation and cDNA synthesis was done by Revert Aid kit (Fermentas, Burlington, Canada). The cDNA products were used for standard RT or Real time PCR. Real time PCR reactions were done by Maxima™ SYBR/ROX qPCR Master Mix (Fermentas, Canada) and monitored in Rotor-gene Q real-time analyzer (Corbet, Australia). The presence or absence of expression of collagen type I was evaluated by RT- PCR (data not shown) and alteration of the level of expression was assessed by RT-PCR in triplicate and then the average threshold cycle was estimated and normalized with GAPDH. The fold change of each target gene was calculated using ΔΔCT method. The primers were designed via primer 3 programs, and complete cDNA sequences obtained from the NIH Gene Bank Entrez program.

***Statistical analysis***

All of the experiments were carried out in triplicate. The data were reported as mean ± SD and statistical significance was analyzed by Student’s t-test. *P*-value≤ 0.05 was deemed statistically significant. 

## Results

***MTT assay***

**Figure 2:**
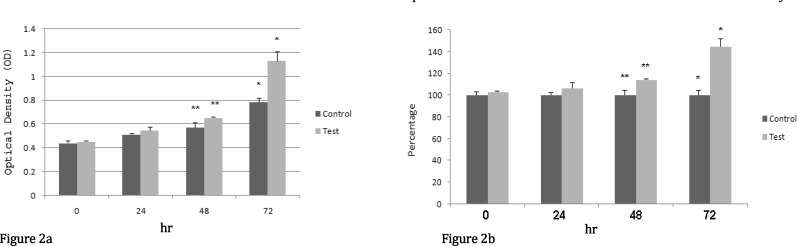
Proliferation of human gingival fibroblasts was analyzed by MTT assay. 5000 initial cells were used. Error bars correspond to mean ±SD. Differences between case and control groups were significant after 48 hr and 72 hr. a: Optical density (OD), b: Cell proliferation (Percentage)

Results of proliferation assay in both case and control groups are illustrated in Figure 2. Gingival fibroblast proliferation assay evaluates the cellular proliferation as a function of mitochondrial activity. 

**Figure 3:**
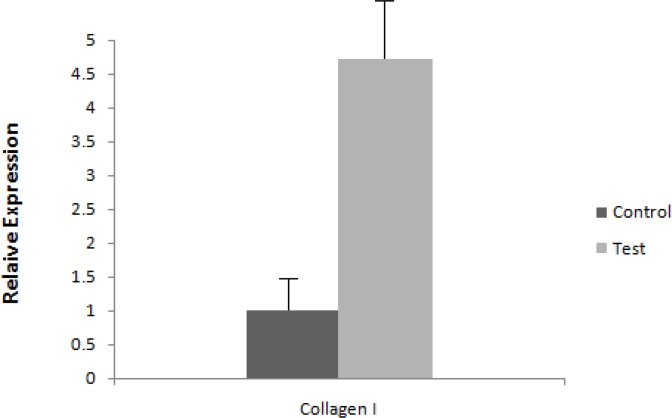
mRNA expression level of collagen I. Expression level is assessed by RT-PCR and results are normalized to control group

Evaluation of cellular proliferation 24 hr after first irradiation revealed that the average absorption was 0.51±0.011 for the control group and 0.54±0.032 for the case group indicating a cell proliferation of 106.5%. On day 2, data revealed that the average absorption was 0.57±0.04 for the control group and 0.65±0.005 for the case group which indicated a significant cell proliferation of up to 114%. Finally after 3 days of irradiation, the average absorption was 0.78±0.37 for the control group and 1.13±0.076 for the case group demonstrating a large increase in cell proliferation to 144.7%.

On day 1, results showed no significant difference in cell proliferation between the case and the control groups. However the differences between the case and the control groups were statistically significant on 48 hr and 72 hr (*P*<0.05) after irradiation. The results of this * in vitro* study revealed that good levels of cell proliferation could be achieved if enough time has been given to the cells to show the effect of laser irradiation on cell proliferation rate.

***RT-PCR***

Either RT or Real time PCR analysis was utilized to evaluate expression of collagen type I as the key gene in collagen production. Figure 3 shows the result for collagen type I expression three days after LLLT. Our data showed that treatment of gingival fibroblasts with laser resulted in an increased collagen type I gene expression on day 3. Therefore there was almost a 5 fold increase in gene expression in the case group compared to the control group. Statistical analysis with REST 2009 software revealed that collagen type I gene expression increased significantly in the case group (*P*< 0.05).

## Discussion

LLLT was first used by Mester *et al* for treatment of chronic ulcers that were not responsive to routine treatments (11). In conservative periodontal strategies, after removal of calculus and microbial plaque, the increasing wound healing capacity would reduce the need for invasive periodontal surgeries. Controlled increase of collagens with specific arrangement in gingival tissue after periodontal surgeries can lead to normal gingival tissue preservation (12). Capacity of gingival connective tissue fibroblasts to produce macromolecules with structural functions especially collagen type I provides an important role for these cells in implantology and periodontology (13). Gingival connective tissue is mainly composed of collagen type I. Elastic fibers and neurovascular elements are also present but in small fractions (14).

Cell culture studies will provide an experimental environment in which the effect of LLLT on gingival fibroblasts can be better evaluated by eliminating many factors that may interfere with clinical trials (15).

Hrnjak *et al* investigated the influence of low-energy laser (He-Ne laser at 632.8 nm) irradiation on fibroblast proliferation. Cultured fibroblasts were irradiated at energy doses of 0.5, 1, 1.5 and 2 J/cm^2^. The results of the study showed that single He-Ne laser irradiation produced a significant stimulation effect on human fibroblast proliferation (16).

Almeida-Lopes *et al* showed that laser irradiation on cells growing in limiting culture conditions would induce cell growth to levels that were observed with cells grown in ideal culture conditions (17). Vinck *et al* found LED (light emitting diode) and LLL irradiation can lead to increased fibroblast proliferation * in vitro* (18). 

Basso *et al* used two different energy doses of LLLT to produce biostimulatory effects on human gingival fibroblast culture. The results showed that LLLT promoted biostimulation of fibroblasts in cell culture at two doses (19). 

Kreisler *et al* have studied the potential stimulatory effect of low-level laser irradiation on the proliferation of human periodontal ligament fibroblasts (PDLF). The outcome of the study showed higher proliferation rate for treatment group compared to control group where the effect was significant at 72 hr post irradiation (20).

The present study revealed that in cultured human gingival fibroblasts photo biomodulation results in significant collagen type I gene expression. There was no significant difference in cell proliferation between the control and the treatment groups at first day post irradiation. However cell proliferation events began after 24 hr and became more significant at 48 and 72 hr post irradiation. Hawkins *et al* confirmed that although ATP increased one hour after low energy laser irradiation, to observe the induction effect of laser on protein production at least 24 hours were required (21).

Furthermore, it has been demonstrated that LLLT on fibroblasts in culture media and animal studies in the process of skin wound healing and bone fracture repair may help in better arrangement of collagen fibers and more compact and parallel fibers after laser photo biomodulation (22). 

## Conclusion

The present study demonstrates that low level laser therapy stimulates human gingival fibroblast (HGF3-PI 53) proliferation and collagen type I gene expression *in vitro* which is in agreement with the results reported on the stimulatory effect of low laser irradiation on gingival fibroblast proliferation *in vitro*.
